# Identifying the factors associated with cesarean section modeled with categorical correlation coefficients in partial least squares

**DOI:** 10.1371/journal.pone.0219427

**Published:** 2019-07-26

**Authors:** Maryam Sadiq, Tahir Mehmood, Muhammad Aslam

**Affiliations:** 1 Department of Mathematics and Statistics, Riphah International University, Islamabad, Pakistan; 2 School of Natural Sciences (SNS), National University of Sciences and Technology (NUST), Islamabad, Pakistan; Mayo Clinic Minnesota, UNITED STATES

## Abstract

Cesarean section (CS) is associated with maternal morbidity and mortality in developing countries. This study is conducted to assess factors associated with CS in Pakistan using partial least squares (PLS) algorithm, where categorical factors are modeled. Nationally representative maternal data from Pakistan Demographic and Health Surveys (PDHS) conducted during 2012-2013 is used in this study. Among correlation coefficient based PLS regression proposed algorithms for categorical factors, Pearson’s Contingency Coefficient (CC) PLS coupled with loading weight (LW) appeared to be the most efficient method in terms of model performance and influential factor selection. Region of residence, type of place of residence, mother’s and her partner’s level of education, wealth index, year of birth, previous terminated pregnancy, use of contraception, prenatal care provided by a doctor and nurse/midwife/LHV (lady health visitor), assistance provided by a nurse/midwife/LHV,number of antenatal visits, size of child, antenatal care provided by government hospital, transport facility for medical care, baby birth status, mother’s age at first birth, preceding birth interval and vaccination of hepatitis B-1 and B2 are found to be significantly affecting the CS delivery method. Correlation coefficient based PLS regression algorithms may serve more efficiently as a multivariate technique to treat high-dimensional categorical data.

## Introduction

Globally, cesarean section (CS) delivery rates have accelerated in recent decades [35, 57]. CS is a surgical technique adopted to prevent medical complications and maternal/fetal mortality during delivery [4]. High quality differential maternal health care facility is a vital necessity for every woman across the world [40]. Unnecessary CS may result in an increased hazard of maternal as well as neonatal deaths [7]. The world health organization (WHO) seriously noticed and evaluated the high CS rate in 2015. Considering two conditions; necessity of CS and avoiding needless CS, WHO recommended to set 5-15% CS rate to rescue the maternal/neonatal lives in essential conditions but avoid unnecessary CS surgery [48].

Pakistan Demographic and Health Survey (PDHS) (2012-13) reported a CS rate of 39% among highly educated women and 34% in women with highest wealth quintile in Pakistan. The final report summerized huge rural-urban variation in CS rates and relatively higher rate for first births (23%), increased antenatal visits (30%) and births in health facility (29%) were observed [13]. According to the WHO report (2015), non-availability and deficiencies of various medical services in developing countries are estimated. The report further documented the highest rate of maternal deaths recorded predominantly in South Asia and Sub-Saharan Africa [47] presenting the adverse maternal and neonatal health outcomes in these regions. Approximately 60% of maternal deaths occur only in 10 countries of the world including Pakistan [47]. China has the highest CS rate among Asian countries [36], while perceptible increase in CS rates is also observed in some South Asian countries in recent years [9].

Diversity of trends in rates and risk factors of CS is noticed predominant over time, especially in Sub-Saharan Africa and South Asia. Regional disparities and disproportionate socioeconomic levels are reported as influential factors of CS [29]. Maternal morbidity is strongly associated with CS in developing countries [2] and specifically elective CS without medical symptoms is reported as a significant risk factor of higher rate of this morbidness [44]. Houweling et al. (2007) examined poor-rich inequalities in maternal care using Demographic and Health Survey (DHS) data from 45 developing countries including Pakistan. They reported huge poor-rich variation in CS rates within rural as well as urban regions [20]. Similarly, two other studies revealed lower CS rates among the poor in developing countries of Africa and South Asia [9, 34]. Another study investigated trends and inequalities in CS rates in Pakistan using data from Pakistan Demographic and Health Surveys (PDHS) administrated during 1990 to 2013. This study documented significant association of CS with wealth index, education and urbanity of women [41]. Olusanya et al. (2009) analyzed data collected during universal newborn hearing screening (UNHS) program in Nigeria. They established significant association of parity, maternal age, maternal positive HIV, social class, lack of antenatal care and multiple gestations with higher risk of emergency CS delivery [45].

Advances in public health generates high-dimensional data having many factors, where some may be irrelevant or redundant. Analyzing such high-dimensional health data faces the curse of dimensionality for effective interpretation of the fitted model. Curse of dimensionality refers to a few samples with many factors which results in multicollinearity and over fitting [26, 27]. In recent years, partial least squares (PLS) based methods have been the subject of increasing concern and attention as a multivariate approach for modeling multi-collinear data. For improved model performance, a large number of modified PLS-based algorithms have been proposed yet. For instance, canonical-powered partial least squares (CPPLS) is established by integrating PLS with canonical correlation analysis for classification and regression problems [23, 24]. Soft-threshold or sparse partial least squares is another version of PLS, introduced [52] by defining a soft-threshold in the algorithm nearly similar to sparse PLS [30]. Other modified PLS algorithms include orthogonal PLS (oPLS) [55], penalized PLS (pPLS)[32, 33], robust PLS (roPLS)[16, 22], kernel PLS (kPLS)[18], interval PLS (iPLS)[43], recursive PLS (rPLS) [19], quadratic PLS (qPLS) [60], generalized PLS (gPLS) [5], weighted PLS (wPLS) [21], genetic algorithm combined with partial least square (gaPLS)[31], radial-based PLS (rbfPLS) [58], distance-based PLS (dbPLS)[28]. Most PLS algorithms deal with factors measured on a continuous scale and no specific algorithms is presented yet to address the categorical scale factors. The main objective of this study is to improve the PLS algorithm to specifically handle the factors measured on categorical scale. The secondary objective is to identify the significant factors associated with CS using a most efficient PLS algorithm. To extend the PLS approach to specifically handle the factors measured on categorical scale, six PLS algorithms with modified loading weights established on categorical measures of association are proposed in this study. The model performance was compared with standard PLS and the algorithms were further used for selecting important factors of CS in Pakistan.

## Materials and methods

### Data set

The data set having 39 factors with 1660 observations is obtained from Pakistan demographic and health survey (PDHS) 2012-13 for the present study. This survey was conducted by the National Institute of Population Studies (NIPS), Pakistan. The United States agency for international development (USAID) provided financial and technical assistance for the survey. The PDHS is part of the worldwide Demographic and Health Survey program, which is designed to collect data on fertility and family planning along with maternal and child health. The delivery method is taken as the response factor (y) with two categories; cesarean section (CS) group and vaginal delivery group having equal observations.

### Partial least square(PLS): Standard form

Ordinary least squares (OLS) modeling is not an appropriate method due to multicollinearity between factors, hence, PLS being an alternative of OLS is used for modeling perspective. Among the several genres of PLS, the orthogonal score PLS algorithm is considered here due to its simplicity and wide applicability in factor selection methods. The algorithm initially centered the data $X_{0}=X-1\bar{x^{\prime }}$ and $y_{0\;}=y-1\bar{y}$. Defined by Naes and Helland [42], it assumes that some A is equal to the number of components to be predicted (where A ≤ p), then for *a* = 1, 2, …, *A* the algorithm runs:

1. Loading weights are computed by
  $$\begin{array}{c} w_{a}=X_{a-1}^{\prime }y_{a-1} \end{array}$$
  The weights define the direction in the space spanned by *X*_*a*−1_ of maximum covariance with *y*_*a*−1_. Loading weights are normalized to have length equal to 1 by
  $$\begin{array}{c} w_{a}⟵w_{a}/\left|\right|w_{a}\left|\right| \end{array}$$
2. Score vector ta is computed by
  $$\begin{array}{c} t_{a}=X_{a-1}w_{a} \end{array}$$
3. X-loadings *p*_*a*_ are computed by regressing the factors in *X*_*a*−1_ on the score vector:
  $$\begin{array}{c} p_{a}=X_{a-1}^{\prime }\frac{t_{a}}{t_{a}^{\prime }t_{a}}. \end{array}$$
  Similarly Y-loadings *q*_*a*_ are computed by
  $$\begin{array}{c} q_{a}=y_{a-1}^{\prime }\frac{t_{a}}{t_{a}^{\prime }t_{a}} \end{array}$$
4. Deflate *X*_*a*−1_ and *y*_*a*−1_ by deducing the contribution of *t*_*a*_:
  $$\begin{array}{c} X_{a}=X_{a-1}-t_{a}p_{a}^{\prime } \end{array}$$
  $$\begin{array}{c} y_{a}=y_{a-1}-t_{a}q_{a} \end{array}$$
5. If *a* < *A* return to 1. The computed loading weights, scores and loadings during each iteration of the algorithm be stored in vectors/matrices which are *W* = [*w*_1_, *w*_2_, …, *w*_*A*_], *T* = [*t*_1_, *t*_2_, …, *t*_*A*_], *P* = [*p*_1_, *p*_2_, …, *p*_*A*_], *q* = [*q*_1_, *q*_2_, …, *q*_*A*_].

The PLS estimators for the regression coefficients for the linear model are found by $\hat{\beta }=W{\left(P^{\prime }W\right)}^{-1}q$ and $\alpha =\bar{y}-\bar{X}\hat{B}$.

The standard PLS works well for quantitative response *y* and explanatory factors from *X* but if response and factors are qualitative, which is the case of the current study, then standard PLS may not be optimal. PLS loading weight plays key role in model building and also has the ability to select influential factors. Loading weights reflect the correlation between response *y* and explanatory factors from *X*. If the data set is qualitative then Cramer’s V, Phi coefficient, Tschuprow’s T coefficient, Contingency Coefficient, Yule’s Q and Yule’s Y are the recommended measures of correlation.

### Cramer’s V (CV) PLS

Cramer’s V correlation coefficient defined by Harald Cramer in 1964 [12] measures the association between nominal factors. It ranges from 0 to 1 and is used to define the PLS loading weights as

$$\begin{array}{c} w_{CV}=\sqrt{\frac{\chi^{2}/n}{min(r-1,c-1)}} \end{array}$$ (1)

Where *χ*^2^ is derived from Pearson’s chi-squared test, *n* is the total number of observations, *r* and *c* denote number of categories in response and factor respectively.

### Phi coefficient (PC) PLS

Phi correlation coefficient also reffered as mean square contingency coefficient [12] is been used in defining the PLS loading weights as

$$\begin{array}{c} w_{PC}=\frac{\chi^{2}}{n} \end{array}$$ (2)

### Tschuprow’s T coefficient (TC) PLS

Tschuprow’s T correlation coefficient [56] is the refined form of Phi coefficient and is used in defining the PLS loading weights as

$$\begin{array}{c} w_{TC}=\sqrt{\frac{\phi^{2}}{\sqrt{\left(r-1)(c-1\right)}}} \end{array}$$ (3)

where *r* and *c* denote the number of categories in response and explanatory factor respectively and *ϕ* is the mean square contingency defined as

$$\begin{array}{c} \phi =\sum_{i=1}^{r}\sum_{j=1}^{c}\frac{{\left(\pi_{ij}-\sum_{j=1}^{c}\pi_{ij}\sum_{i=1}^{r}\pi_{ij}\right)}^{2}}{\sum_{j=1}^{c}\pi_{ij}\sum_{i=1}^{r}\pi_{ij}} \end{array}$$ (4)

Where *ϕ*_*ij*_ is the proportion of the sample in the (*i*, *j*)^*th*^ cell of the *r* × *c* contingency table.

### Pearson’s contingency coefficient (CC) PLS

Pearson’s contingency coefficient [15] measures the strength of association between categorical factors, and is used for defining the loading weights as

$$\begin{array}{c} w_{CC}=\sqrt{\frac{\chi^{2}}{N+\chi^{2}}} \end{array}$$ (5)

### Yule’s Q (YQ) PLS

Yule’s Q correlation coefficient [62] determines the strength of relationship between the expalnatory factor and the response. Yule’s Q based loading weights are defined as;

$$\begin{array}{c} w_{YQ}=\frac{OR-1}{OR+1} \end{array}$$ (6)

where OR represents the odds ratio.

### Yule’s Y (YY) PLS

Yule’s Y or the coefficient of colligation [62] is a measure of association for qualitative data.

$$\begin{array}{c} w_{YY}=\frac{\sqrt{OR}-1}{\sqrt{OR}+1} \end{array}$$ (7)

### Filter methods for factor selection in PLSR

In standard PLS a variety of factor selection methods exist [38, 53]. Here the following five filter methods for subset selection of influential explanatory factors are considered.

### Loading weight(LW)

The loading weighs *r*_*j*_ used as a measure of identification of important factor is defined as [37];

$$\begin{array}{c} LW=|\frac{w_{a,j}}{max(w_{a})}| \end{array}$$ (8)

### Regression coefficients(RC)

The PLS estimator of the regression coefficient for the model is represented by;

$$\begin{array}{c} RC=W{\left(P^{\prime }W\right)}^{-1}q. \end{array}$$ (9)

### Variable importance in projection (VIP)

Variable importance in projection defined by [14, 59] is the measure to assemble the importance of each factor based on loading weight. For factor j, the VIP measure is

$$\begin{array}{c} VIP=\sqrt{p\sum_{a=1}^{A}[SS_{a}\left(w_{aj}/\|w_{a}{\left\|\right)}^{2}\right]}/\sum_{a=1}^{A}\left(SS_{a}\right) \end{array}$$ (10)

where *SS*_*a*_ denote the sum of squares explained by the *a*^*th*^ component and the importance of *j*^*th*^ factor is represented by the term (*w*_*aj*_/‖*w*_*a*_‖)^2^]. Hence, the VIP score *V*_*j*_ represents the contribution of *j*^*th*^ factor based on variance explained by each component. If *V*_*j*_ is less than a defined threshold, *j*^*th*^ factor can be excluded, where the threshold ranges from 0 to ∞. A threshold between 0.83 to 1.21 is recommended [11] while *V*_*j*_ > 1 is a generally accepted threshold [14, 17].

### Selectivity ratio (SR)

The selectivity ratio (SR) is the ratio between explained variance (*V*_*e*_) and residual variance (*V*_*r*_) for *i*^*th*^ factor on target-projected component for reponse. SR is defined as;

$$\begin{array}{c} SR=\left(V_{ej}\right)/\left(V_{rj}\right)j=1,2... \end{array}$$ (11)

The defined threshold is *SR* > *F*(*critical*) where *F*(*critical*) represents the value corresponding to the F-test. Hence, the factor with SR value greater than the threshold is included in the model. The SR provides the numerical contribution of each factor included in the model. The higher the value of SR, the more important the factor is, for prediction purpose. Lowest SR recommends to eliminate the corresponding factors without affecting the performance [51].

### Significance multivariate correlation (SMC)

The basic concept of significance multivariate correlation is to minimize the influence of irrelevant factors in X-structure and enhance the importance of factors which have high contribution related to response factor. SMC can be used for simulated as well as real data sets.

$$\begin{array}{c} SMC=MS_{Regression}/MS_{Residual} \end{array}$$ (12)

Where *MS*_*Regression*_ is the mean square regression and *MS*_*Residual*_ is denotes the mean square residual [54]

## Results

**Fig 1:**
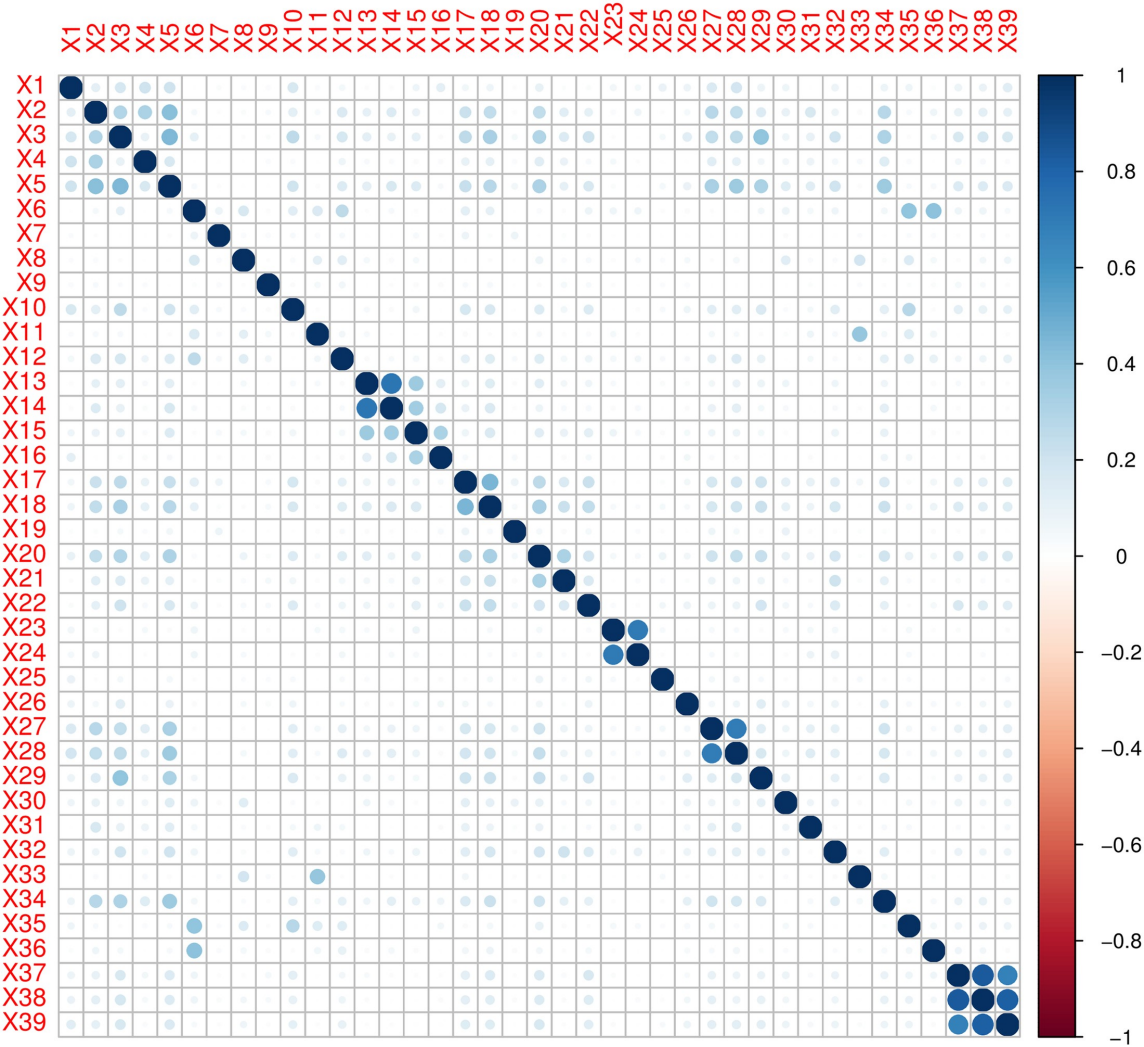
Correlogram by Cramer’s V correlation matrix is presented in upper panel while the lower panel represented the Phi correlation matrix. Color intensity and the size of the circle are proportional to the strength of the correlation measure between factors.

The CS data set contains 39 factors sampled over 1660 samples (mothers). Cramer’s V and Phi correlation coefficients are used to detect the presence of multicollinearity in the nominal data. The correlograms shown in Fig 1 evidenced strong correlation between 12 factors while moderate correlation is observed between various other factors by both methods. Presence of multicollinearity violates the assumption of linear independence and hence, logistic regression and generalized linear models become inappropriate to handle collinear data. Therefore, PLSR is used to deal categorical data with high multicollinearity.

**Fig 2:**
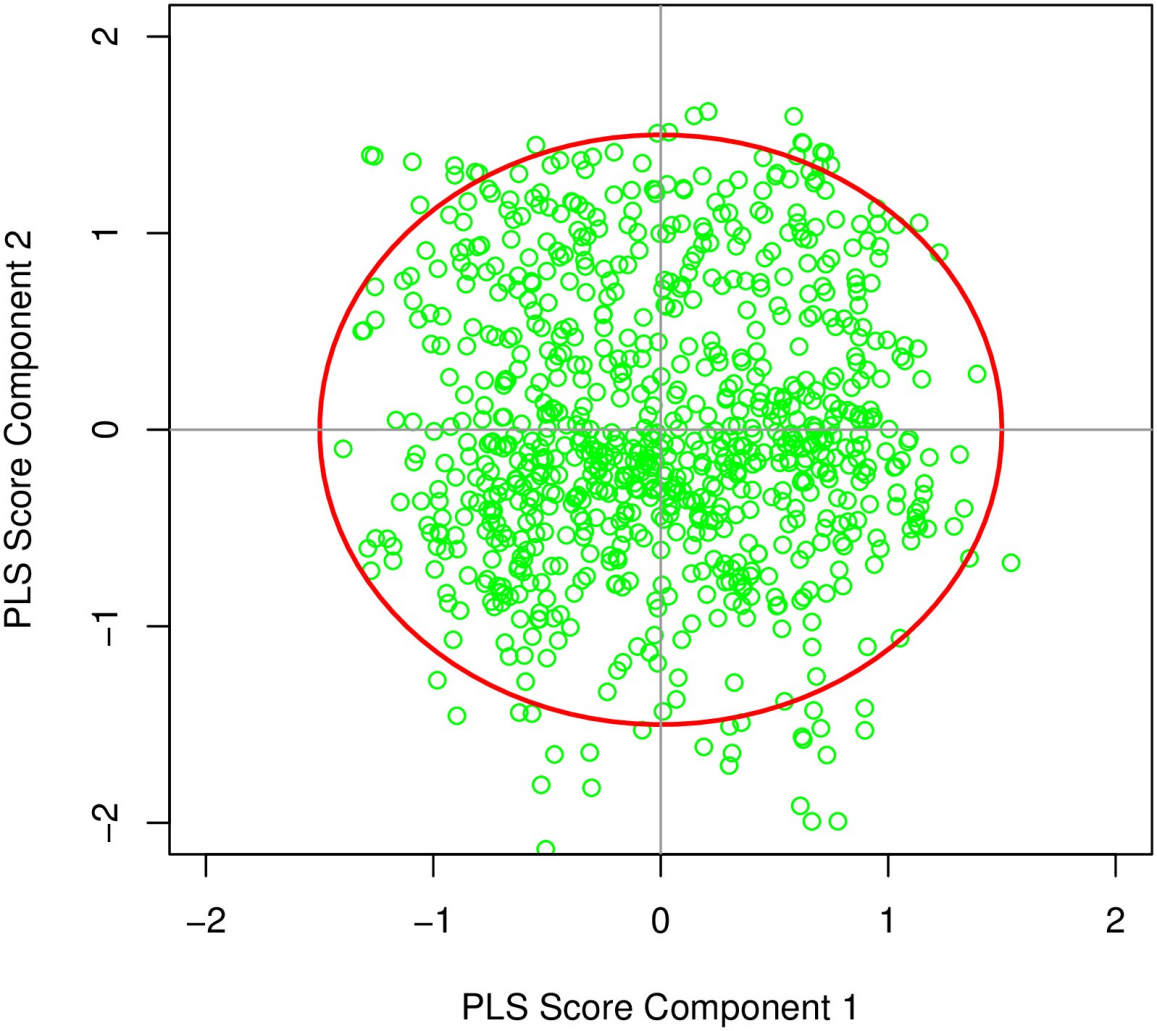
The PLS scores from component 1 and component 2 were plotted in the upper panel. Mothers laying out of red circle were considered outliers. For illustration purposes, the visualized graph showing several samples (mothers) grouped in one cluster is presented in the lower panel.

The survey data may include some noise samples. It is important to identify and eliminate the noise samples. For this, the standard PLS model over the data is fitted and PLS scores from component 1 and component 2 were plotted, as presented in the upper panel of Fig 2. The women laying out of red circle were supposed to be outliers and were discarded from the data set for further analysis. For model fitting, samples are required to be independent,therefore, the PLS scores were clustered. For illustration purpose, lower panel of Fig 2 presents the visualized graph showing several samples (mothers) grouped in one cluster. The samples grouped in a cluster are correlated, hence one member from each cluster should be considered only. Since the samples/mothers can be divided into two groups, namely CS group and vaginal delivery group. Both groups are clustered separately through k-means and optimum number of clusters were found. Therefore, 100 women from CS group and 100 women from the vaginal delivery group were selected by picking the centroid of each cluster.

**Fig 3:**
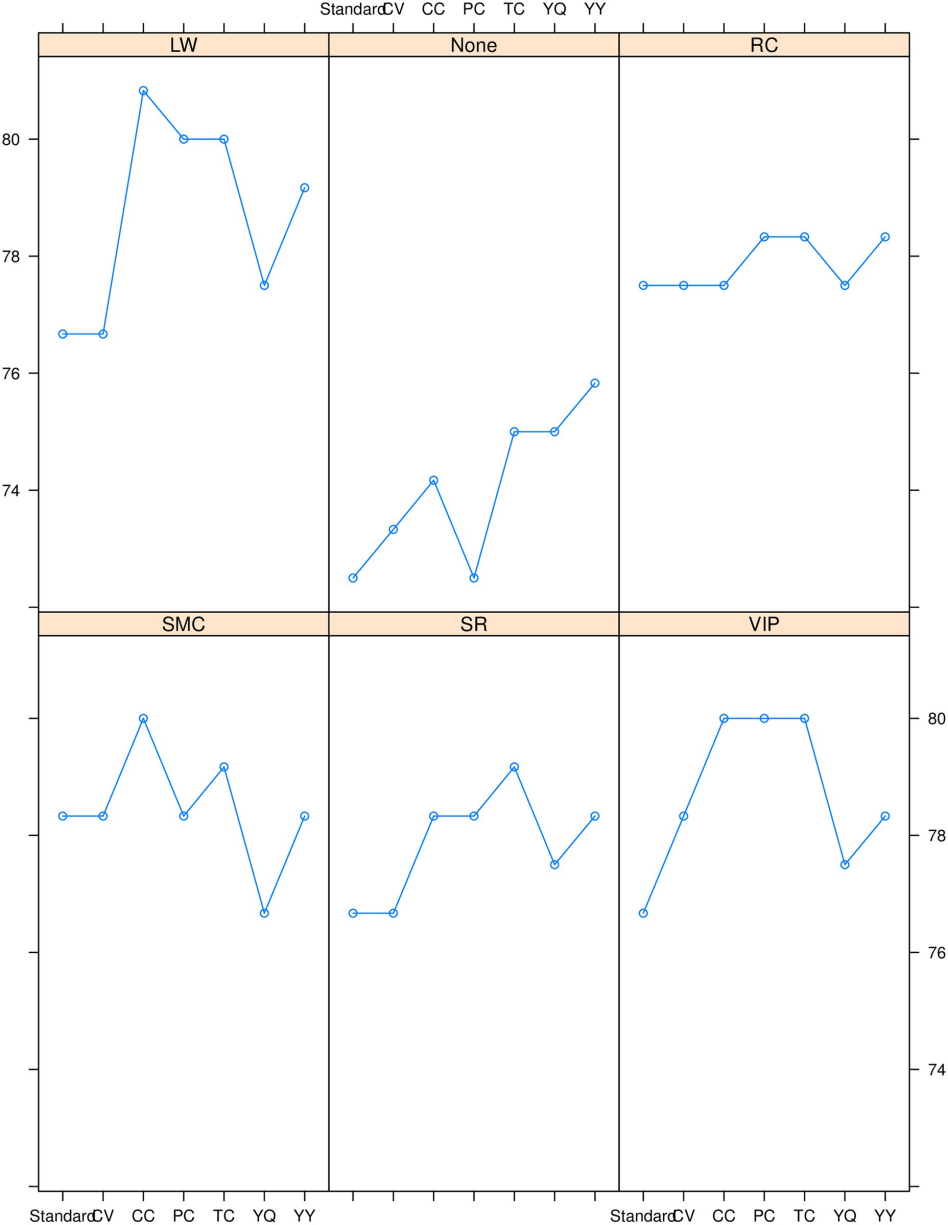
The validation accuracy of PLS methods including, Cramer’s V PLS (CV-PLS), Phi coefficient PLS (PC-PLS), Tschuprow’s T coefficient PLS (TC-PLS), Pearson’s contingency coefficient PLS (CC-PLS), Yule’s Q PLS (YQ-PLS) and Yule’s Y PLS (YY-PLS) models against the filter subset selection methods including LW, RC, VIP, SR and SMC by using lattice plot is presented in the upper panel, while the calibration accuracy is presented in the lower panel.

After initial processing, 39 explanatory factors measured over 200 samples (mothers) were considered for further analysis. To have a reliable model performance, the data was split into training (70%) and test data (30%). The fitted model was trained over the training data, while the model performance was measured over the test data. The split of the data into training and test was done randomly. To measure reliability and accuracy of different PLS models, validation and calibration of the proposed methods are being observed. Model validation over test data and model calibration over training data were measured for all PLS algorithms with and without filter factor selection methods to compare the discriminant accuracy of new and existing PLS methods. In order to remove the effect of randomness the data was split 10 times, in each split the model was trained on training data and was evaluated on test data by computing validation and calibration accuracy. Six PLS based models called Cramer’s V PLS (CV-PLS), Phi Coefficient PLS (PC-PLS), Tschuprow’s T Coefficient PLS (TC-PLS), Pearson’s Contingency Coefficient PLS (CC-PLS), Yule’s Q PLS (YQ-PLS) and Yule’s Y PLS (YY-PLS) are proposed and compared them with standard PLS through validation and calibration. Each PLS method is evaluated through five filter subset selection methods, including loading weights (LW), regression coefficients (RC), variable importance in projection (VIP), selectivity ratio (SR) and significance multivariate correlation (SMC) for factor selection.The validation accuracy of all PLS methods with and without factor selection methods is presented in the upper panel of Fig 3.

The plot for standard PLS without filter method is presented as ‘None’ in Fig 3. It indicates that the average validation performance of five introduced PLS algorithms is higher than the standard PLS without any filter measure while PC-PLS exhibits similar performance as standard PLS. All newly introduced PLS regression algorithms combined with LW, VIP and SR filter methods also showed higher validation performance than standard PLS regression combined with these filter methods. Equal accuracy of CV-PLS, CC-PLS, YQ-PLS and standard PLS is observed for RC filter method. Interestingly, it is noted that only YQ-PLS combined with SMC showed lower performance than standard PLS combined with same filter method. The CC-PLS combined with LW showed highest validation accuracy in differentiating the two classes of mothers.

The calibration accuracy of all PLS methods combined with filter methods is presented in the lower panel of Fig 3. In case of calibration performance all proposed PLS algorithms combined with RC and SR filter methods and also without considering any filter method improved the accuracy of dealing categorical variables than standard PLS with same condition of factor selection measures.It is observed that the CC-PLS algorithm combined with a LW factor selection method appears to be most efficient amongst all other methods having highest median validation performance and hence, considered for further analysis.

To strengthen these findings, analysis of variance test was conducted where the significance of PLS methods and factor selection measures were assessed in explaining the variation in accuracy of the models. Anova results are presented in Table 1. This indicates that the CC-PLS has ≈ 24% accuracy in differentiating the CS group, which is 2.39% more compared to standard PLS with (*p* < 0.001). Similarly LW factor selection method has ≈ 30% accuracy for differentiating the CS group, which is 5.29% more compared without selection measure (*p* < 0.001). Hence CC-PLS coupled with LW is being applied for further analysis of CS group and selection of influential factors.

**Table 1:** Anova results showing the significance of PLS methods and factor selection measures in explaining the variation in accuracy of the models are presented.

	Estimate	Standard error	P-value
*FactorSelection*			
*LW*	5.29	0.70	<0.001
*RC*	3.904	0.70	<0.001
*SMC*	4.60	0.70	<0.001
*VIP*	4.52	0.70	<0.001
*SR*	4.55	0.70	<0.001
*PLSmethod*			
*CC*—*PLS*	2.39	0.76	0.001
*CV*—*PLS*	0.64	0.76	0.399
*PP*—*LS*	1.08	0.76	0.15
*TT*—*PLS*	2.67	0.76	0.001
*YQ*—*PLS*	1.22	0.76	0.11
*YY*—*PLS*	2.33	0.76	0.002

For modeling the dataset, CC-PLS coupled with LW was executed and coefficients are presented in Table 2 where inflectional factors are extracted.

**Table 2:** CC-PLS coefficients are presented where inflectional factors are extracted by coupling CC-PLS with LW.

Factor	Coefficient
Region of residence	-0.250
Type of place of residence	-0.237
Mother’s educational level	0.114
Wealth index	0.056
Year of birth	0.056
Ever had a terminated pregnancy	-0.090
Used any contraceptive method	0.107
Prenatal care by a doctor	0.092
Prenatal care by a nurse/midwife/LHV	-0.089
Assisted by a nurse/midwife/LHV	-0.072
Number of antenatal visits	0.288
Size of child at birth	0.199
Antenatal care provided by government hospital	-0.088
Transportation for medical facility	0.135
Partner’s educational level	-0.027
Baby birth status	0.056
Mother’s age at first birth	0.102
Preceding birth interval	0.092
Received vaccination hapititis B-1	0.232
Received vaccination hapititis B-2	0.083

After analysis, 20 influential factors which best differentiate the CS group and vaginal delivery group were found. The negative association of region and type of place of residence with the CS group showed that for every additional unit in region and type of residence, the CS group decreased by an average of 0.250 and 0.237 units respectively. A significant positive association of mother’s education level with CS method is observed demonstrating 0.114 unit increase in CS group due to this factor. On the other hand, negative association of mother’s partner education level is observed. Wealth index and year of birth are observed to be positively associated with the CS group showing an average increase of 0.056 units. The results further demonstrate that the unit change in earlier terminated pregnancy decreases the CS group by 0.09 units and contraceptive use increase the CS group by 0.107 units. CS group is expected to decrease by 0.089 units by a unit change in prenatal care by nurse/midwife/LHV while positive association of size of the child at the time of birth with delivery method is observed showing 0.199 unit change in CS group by a unit increase in this factor. Furthermore, if assistance given by a nurse/midwife/LHV changes by one unit, CS group decreased by 0.072 units. Prenatal care provided by a doctor increases the CS group by 0.092 units. Antenatal care provided by government hospital is negatively associated with CS group and availability of transport facility is positively associated with this group. New born birth status and preceding birth interval are found to be positively associated with CS group. CS group is predicted to increase by 0.102 units when the mother’s age at first birth goes up by one respectively. The present study found that vaccination of Hepatitis B-1 and B-2 grows up the CS group by 0.208 and 0.264 units respectively, but no previous study was found in this context.

## Discussion

This study identified the factors associated with CS using a representative sample data extracted from Pakistan demographic and health survey (PDHS) 2012-13. Presence of multicollinearity prompted the use of PLS as one of the popular substitute of linear regression. Data is processed for elimination of outliers and clustering through k-means before further analysis. The resulting sample is then split randomly into test and training data sets. Six PLS algorithms based on correlation coefficients are proposed to specifically deal the categorical factors and compared with standard PLS to evidence the improvement in model building. The proposed algorithms include Cramer’s V PLS (CV-PLS), Phi Coefficient PLS (PC-PLS), Tschuprow’s T Coefficient PLS (TC-PLS), Pearson’s Contingency Coefficient PLS (CC-PLS), Yule’s Q PLS (YQ-PLS) and Yule’s Y PLS (YY-PLS). Furthermore, five well-known filter based subset factor selection measures were incorporated with each PLS algorithm and then, compared with standard PLS to observe variation in the efficiency of proposed and existing PLS algorithms with and without filter selection measures. The filter based subset factor selection measures considered in this study are; loading weights (LW), regression coefficients (RC), variable importance in projection (VIP), selectivity ratio (SR) and significance multivariate correlation (SMC).

Validation and calibration accuracy is measured over 10 iterations to compare the performance of seven PLS algorithms with and without filter selection measures.

Regarding validation and calibration accuracy, two important and interesting facts are observed. Firstly, without considering any filter-based factor sub-set selection method, CV-PLS, TC-PLS, CC-PLS, YQ-PLS, YY-PLS evidenced improved validation performance compared to standard PLS for dealing categorical factors. This significant improvement suggested application of proposed PLS algorithms for model building specifically managing such type of data. While PC-PLS showed equal performance as standard PLS for validated data without filter measure. This uniformity in efficiency supported PC-PLS to be an alternative choice of standard PLS in the specific case of categorical response factor. All proposed PLS algorithms reflected higher accuracy compared to standard PLS for calibrated data without any filter measure. The higher calibration performance showed increased reliability and accuracy of proposed PLS algorithms. Secondly, and more significantly, increased efficiency is observed for all PLS algorithms combined with factor selection measures compared to without these measures for validated as well as calibrated data. Overall, the proposed PLS algorithms with and without factor selection measures enhanced the accuracy for validated and calibrated data compared to standard PLS with and without these measures, respectively. For current data set, the CC-PLS algorithm combined with LW factor selection measure is observed to be most efficient model amongst all other models having highest median validation accuracy performance.

The CC-PLS coupled with LW was recommended for modeling the dataset and 20 influential factors are observed to identify the CS group. The association of region and type of place of residence with CS group is observed for the present data. A study using the data of 150 countries consistently evidenced that developed regions have the highest rate of CS [8]. Another study conducted in Bangladesh showed that place of residence was an important predictor of CS for childbirth [25]. A significant association of mother’s and her partner’s education level with CS group is identified. Along with parent’s education, wealth index and year of birth are also observed to be associated with CS group. Previous studies evidenced that parent’s level of education and wealth index effected the CS rates [6, 10, 61].

Among factors related to pregnancy history, mother’s age at first birth, preceding birth interval, earlier terminated pregnancy and contraception were found associated with the CS group for the current study. Results of other studies that investigated the relationship of terminated pregnancy history, use of contraceptive methods, mother’s age and birth intervals with CS ratio were consistent with the present study [1, 3, 49, 50]. Regarding maternal care factors, prenatal care provided by a doctor and nurse/midwife/LHV, assistance given by a nurse/midwife/LHV, antenatal care provided by government hospital and availability of transport facility to get medical help are evidenced to be related to identify the CS group. Concerning child related factors, the present data established association of new born birth status and size of the child at the time of birth with CS group. Several other studies pointed the association of cesarean section with prenatal care, facilities and antenatal visits. Moreover, significant association between CS delivery method and newborn status, weight, size and head circumference was also reported previously [1, 39, 46, 49]. The present study found that vaccination of Hepatitis B-1 and B-2 are significantly associated with CS group, but no previous investigation was found in this context.

## Conclusion

Proposed PLS algorithms were a better choice regarding model performance and factor selection of categorical health data. It indicates that these correlation coefficients based algorithms produce models with superior interpretation potential. Using CC-PLS with LW, the factors identified as the significant predictors of CS were commensurate with other studies. So, correlation coefficient based PLS regression algorithms have the potential as a multivariate technique in public health research to treat high-dimensional categorical data more efficiently.

## Supporting information

S1 File Variable description. Complete description of response and explanatory factors including each category is presented.
(DOCX)

S2 File Notes on DHS data sets. Information about data sets, questionnaires, codes and data files is presented.
(DOC)

S3 File Minimal data set. Original data set having first 50 observations is provided to replicate the study findings.
(SAV)
