# Correction: Identifying the factors associated with cesarean section modeled with categorical correlation coefficients in partial least squares

**DOI:** 10.1371/journal.pone.0221955

**Published:** 2019-08-28

**Authors:** The PLOS ONE Staff

Figs 1, 2 and 3 only partially appear. Please see the correct Figs 1, 2 and 3 here. The publisher apologizes for the errors.

**Fig 1:**
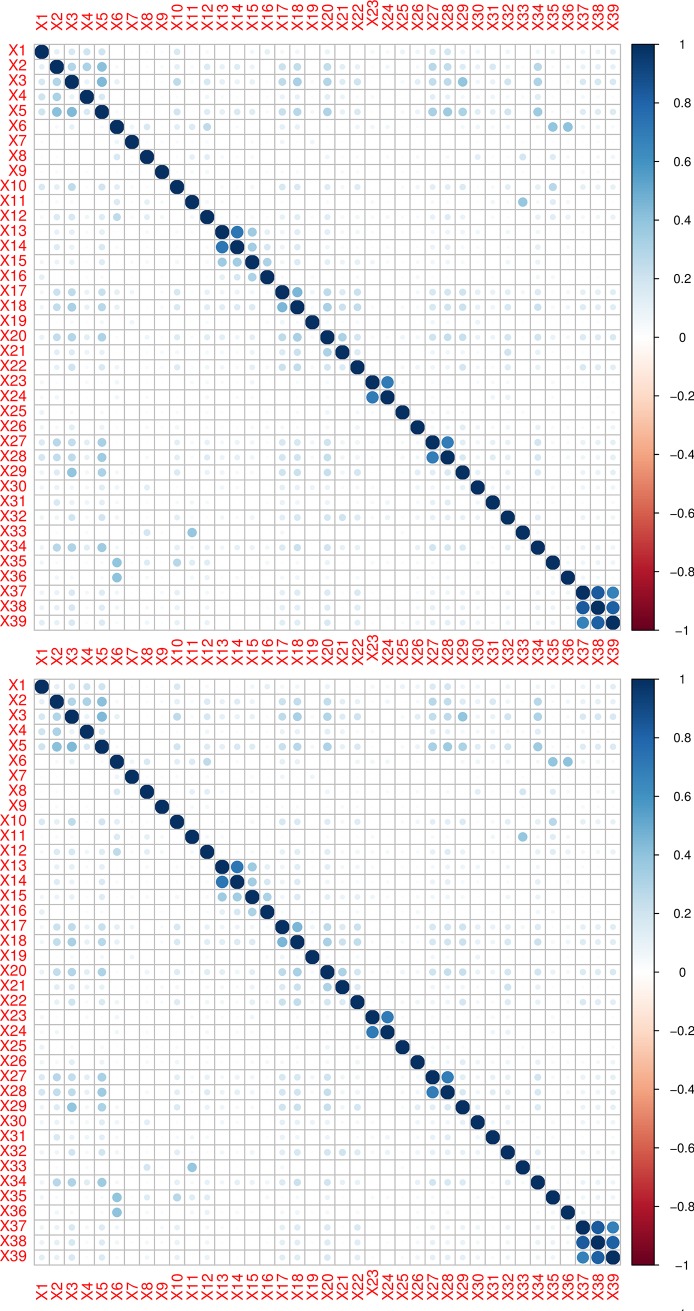
Correlogram by Cramer’s V correlation matrix is presented in upper panel while the lower panel represented the Phi correlation matrix. Color intensity and the size of the circle are proportional to the strength of the correlation measure between factors.

**Fig 2:**
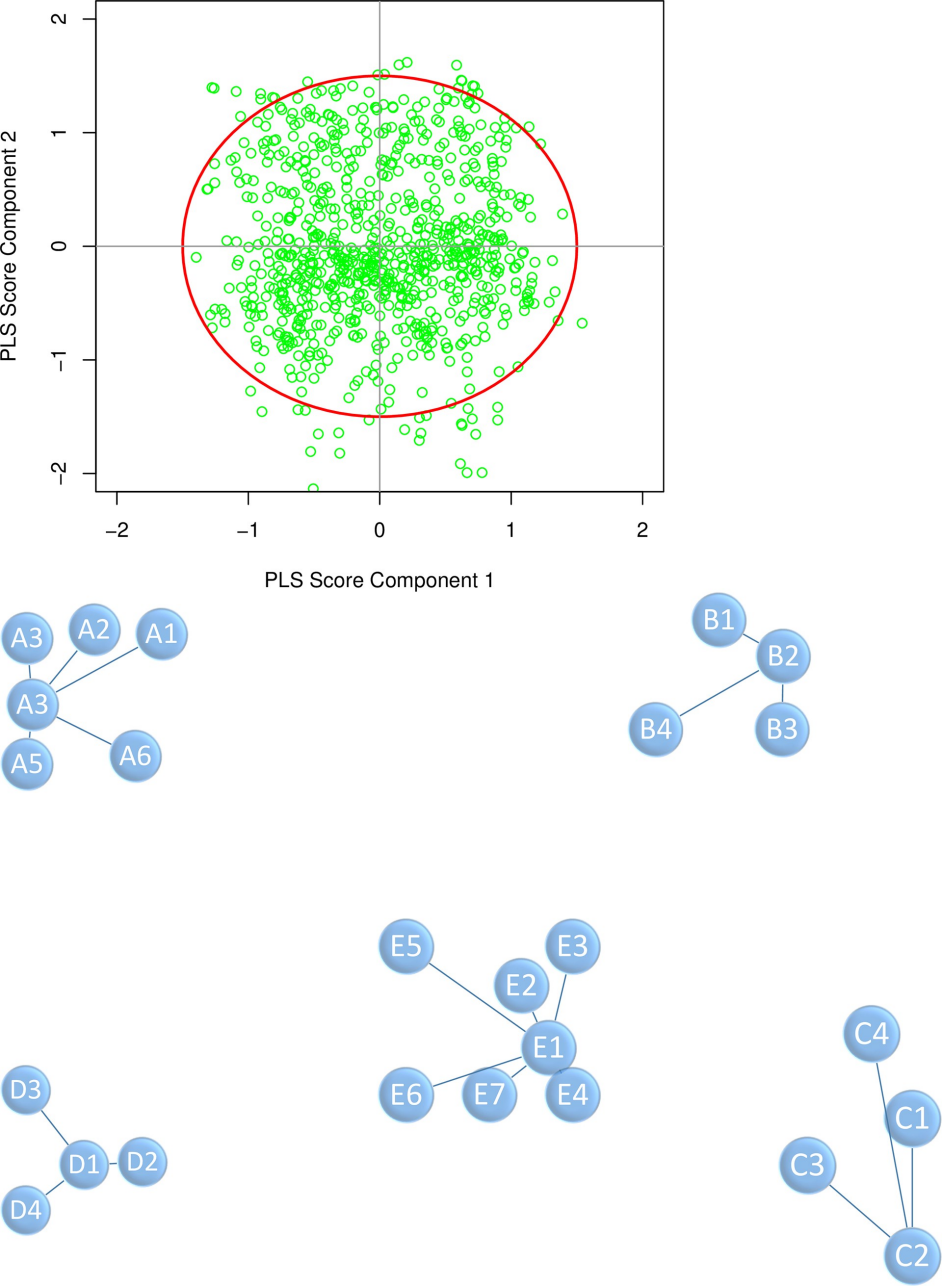
The PLS scores from component 1 and component 2 were plotted in the upper panel. Mothers laying out of red circle were considered outliers. For illustration purposes, the visualized graph showing several samples (mothers) grouped in one cluster is presented in the lower panel.

**Fig 3:**
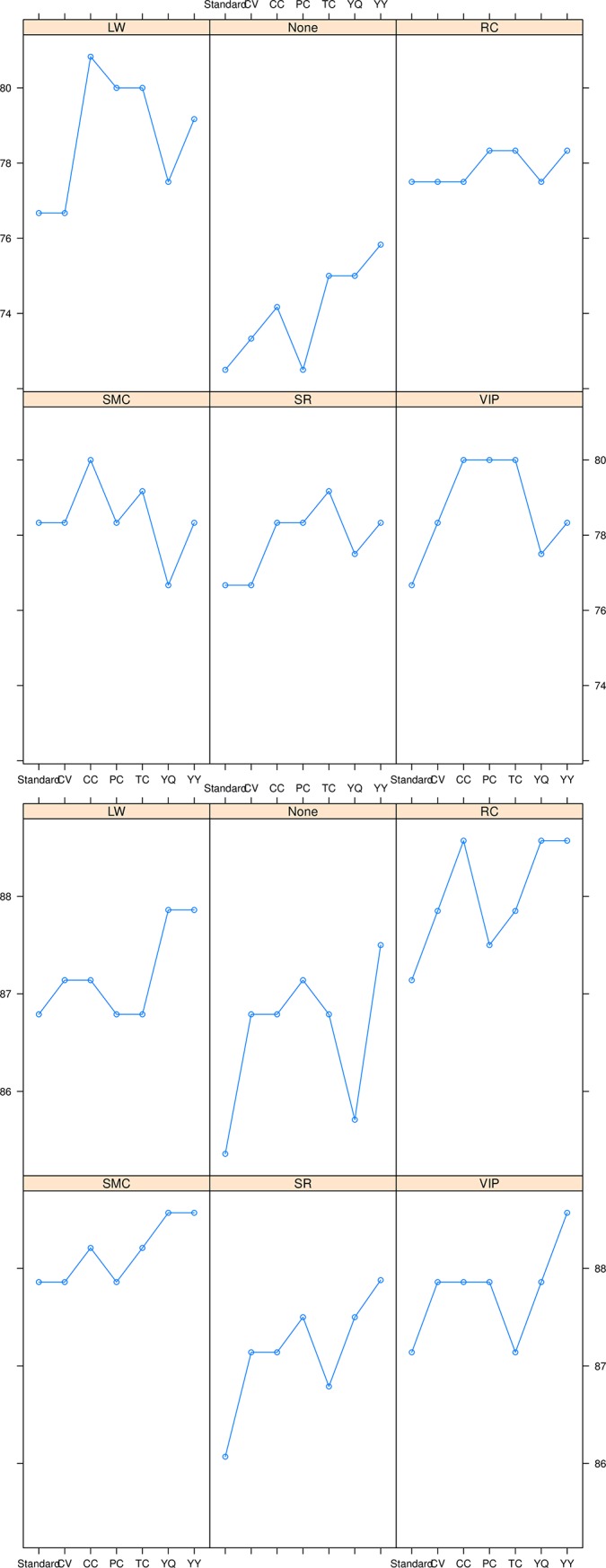
The validation accuracy of PLS methods including, Cramer’s V PLS (CV-PLS), Phi coefficient PLS (PC-PLS), Tschuprow’s T coefficient PLS (TC-PLS), Pearson’s contingency coefficient PLS (CC-PLS), Yule’s Q PLS (YQ-PLS) and Yule’s Y PLS (YY-PLS) models against the filter subset selection methods including LW, RC, VIP, SR and SMC by using lattice plot is presented in the upper panel, while the calibration accuracy is presented in the lower panel.
