# Supplementary material for: Identifying the factors associated with cesarean section modeled with categorical correlation coefficients in partial least squares
Source: PLoS One. 2019 Jul 26;14(7):e0219427. doi: 10.1371/journal.pone.0219427 (PMC6660071; doi:10.1371/journal.pone.0219427)
Supplement: S1 File — Complete description of response and explanatory factors including each category is presented. (DOCX) [file pone.0219427.s001.docx]

| S/No. | Response | Variable | Description | Categories |
| --- | --- | --- | --- | --- |
|  | Y= Delivery Method (cesarean section group and vaginal delivery group) | X1 | Region | - 1 = "Punjab" - 2 = "non-Punjab" |
|  |  | X2 | Type of place of residence | - 1 = "Urban" - 2 = "Rural" |
|  |  | X3 | Highest educational level of respondent | - 0 = "Illiterate" - 1 = "Literate" |
|  |  | X4 | Source of drinking water | - 0 = "Piped Water" - 1 = "Other" |
|  |  | X5 | Wealth index | - 0 = "Poor" - 1 = "non-poor" |
|  |  | X6 | Pregnancy order | - 0 = "Primiparity" - 1 = "multiparity" |
|  |  | X7 | Child is twin | - 0 = "Single birth" - 1 = "multiple births" |
|  |  | X8 | Year of birth | - 0 = "2007-2010" - 1 = "2011-2013" |
|  |  | X9 | Sex of child | - 0 = "Female" - 1 = "Male" |
|  |  | X10 | Age of respondent at 1st birth | - 0 = "Less than or equals to 20" - 1 = "greater than 20" |
|  |  | X11 | Ever had a terminated pregnancy | - 0 = "No" - 1 = "Yes" |
|  |  | X12 | Ever use any contraceptive method | - 0 = "Never used" - 1 = "Used" |
|  |  | X13 | Prenatal: doctor | - 0 = "No" - 1 = "Yes" |
|  |  | X14 | Prenatal: Nurse/midwife/LHV | - 0 = "No" - 1 = "Yes" |
|  |  | X15 | Assistance: doctor | - 0 = "No" - 1 = "Yes" |
|  |  | X16 | Assistance: nurse/midwife/LHV | - 0 = "No" - 1 = "Yes" |
|  |  | X17 | Timing of 1st antenatal check (months) | - 0 = "first trimester" - 1 = "After first trimester" |
|  |  | X18 | Number of antenatal visits during pregnancy | - 0 = "less than 4" - 1 = "greater than or equals to 4" |
|  |  | X19 | Size of child at birth | - 0 = "Average" - 1 = "Not average" |
|  |  | X20 | During pregnancy: weighed | - 0 = "No" - 1 = "Yes" |
|  |  | X21 | During pregnancy: blood pressure taken | - 0 = "No" - 1 = "Yes" |
|  |  | X22 | During pregnancy, given or bought iron tablets/syrup | - 0 = "No" - 1 = "Yes" |
|  |  | X23 | Antenatal care: government hospital | - 0 = "No" - 1 = "Yes" |
|  |  | X24 | Antenatal care: private hospital/clinic | - 0 = "No" - 1 = "Yes" |
|  |  | X25 | Drugs for intestinal parasites during pregnancy | - 0 = "No" - 1 = "Yes" |
|  |  | X26 | Use tobacco | - 0 = "No" - 1 = "Yes" |
|  |  | X27 | Getting medical help for self: distance to health facility | - 0 = "Not a big problem" - 1 = "Big problem" |
|  |  | X28 | Transportation for medical facility | - 0 = "Not a big problem" - 1 = "Big problem" |
|  |  | X29 | Husband/partner's education level | - 0 = "Illiterate" - 1 = "Literate" |
|  |  | X30 | Respondent's occupation | - 0 = "Not working" - 1 = "working" |
|  |  | X31 | Blood relation with husband | - 0 = "No" - 1 = "Yes" |
|  |  | X32 | During pregnancy: have ultrasound exam | - 0 = "No" - 1 = "Yes" |
|  |  | X33 | Baby birth status | - 0 = "Born dead or lost before full term" - 1 = "Born alive" |
|  |  | X34 | Media Exposure | - 0 = "No" - 1 = "Yes" |
|  |  | X35 | Women age at the time of birth | - 0 = "15-25 years" - 1 = "26-48 years" |
|  |  | X36 | Preceding birth interval (months) | - 0 = "less than or equals 36 months" - 1 = "First birth or greater than 36 months" |
|  |  | X37 | Received hepatitis B-1 | - 0 = "No" - 1 = "Yes" |
|  |  | X38 | Received hepatitis B-2 | - 0 = "No" - 1 = "Yes" |
|  |  | X39 | Received hepatitis B-3 | - 0 = "No" - 1 = "Yes" |
