# Supplementary material for: Identifying the factors associated with cesarean section modeled with categorical correlation coefficients in partial least squares
Source: PLoS One. 2019 Jul 26;14(7):e0219427. doi: 10.1371/journal.pone.0219427 (PMC6660071; doi:10.1371/journal.pone.0219427)
Supplement: S2 File — Information about data sets, questionnaires, codes and data files is presented. (DOC) [file pone.0219427.s002.doc]

**NOTES ON DHS DATASETS**

DHS process the data in a way that brings information ‘down’ from each higher analytical level in the data, to the lower levels.  Thus household information about water source, toilet facilities, cooking facilities, Wealth Index, are attached to the woman’s, men’s and children’s files; as this household information may have relevance for analysis of the woman, man, or child.  Likewise mother’s information such as education, marital status, height and weight, defacto/dejure status, etc, is attached to each of her children, as this is potentially relevant to the analysis of them. In DHS surveys there are multiple units of analysis, and these in turn are interrelated.  Great care is taken to assure that these relations and their linkages are complete and correct.

The three core questionnaires in ***DHS*** surveys are:  the Household Questionnaire, the Women’s Questionnaire, and the Men’s questionnaire. There are also several standardized modules for countries with interest in other topics, such as malaria, domestic violence or maternal mortality. All additional modules are incorporated into the Household, Women’s, or Man’s questionnaires. Since the very beginning of DHS a recode file was designed for the sake of consistency and comparability across surveys. In the first phase of the DHS (DHS-I) the recode was defined only for the Women’s Questionnaire. The recode file proved to be very useful and as a result since DHS-II, a recode file was introduced for the Household and the Men’s questionnaires.

Recode files are initially created using a hierarchical model and later exported to flat files. There are two physical recode hierarchical data files. The first one includes the Household and Women’s Questionnaire and the second one is for the Men's Questionnaire. The hierarchical data file is broken down into a number of records. The records were originally designed to map different sections of the model questionnaires, but because of changes among phases that is not the case anymore. Some of these records are repeating or multiple-occurrence records while others are single-occurrence records. Single records contain simple, single-answer variables. Multiple records are used to represent sets of questions that are repeated for a number of events.

There are special records to keep variables that are not part of the model questionnaires but were included in a particular country. These records are known as country-specific records and they can also be multiple or single depending on whether the question was added to a single or multiple section in the questionnaire.

The **types of datasets** generated for each survey vary by survey design; however there are seven common types of recode data files associated with the core questionnaires.  The three   questionnaires: the household, the woman’s and the men’s; from an analytical point of view, contain the analytical units of: household information, household member’s information, women’s information, children’s information (of the interviewed women), and men’s information.  Further the children’s information exists in two groups – basic data for all children of a woman, and more in depth information for children born in the last five years.  And then, were possible, individual men and woman are matched into couples.  And if an HIV test was done, there are the HIV results.  Thus, in practice, there are 8 data files:

-         Household file (HR)
-         Household members, or persons file (PR)
-         Women’s file (IR)
-         All Births file (BR)
-         Children born in the 5 years prior to the interview, or kids file (KR)
-         Men’s file (MR)
-         Couple’s file (CR)
-         HIV file (AR)

**Household Data - Household Recode (HR)** 
This dataset has one record for each household. It includes household member's roster but no information from the individual women/men questionnaires is present in this file. The unit of analysis (case) in this file is the household.
 

**Household Listing Data - Household Member Recode (PR)** 
This dataset has one record for every household member. It includes variables like sex, age, education, orphan hood, height and weight measurement, hemoglobin, etc. It also includes the characteristics of the households where the individual lives. The unit of analysis (case) in this file is the household member.
 
**Individual Woman's Data - Individual Recode (IR)** 
This dataset has one record for every eligible woman as defined by the household schedule. It contains all the data collected in the woman's questionnaire plus some variables from the household. Up to 20 births in the birth history, and up to 6 children under age 5, for whom pregnancy and postnatal care as well as immunization and health data were collected, can be found in the file. The fertility and mortality programs distributed by DHS use this file for data input. The unit of analysis (case) in this file is the woman.
 
**Man's Data - Male Recode (MR)** 
This dataset has one record for every eligible man as defined by the household schedule. It contains all the data collected in the man's questionnaire plus some variables from the household. The unit of analysis (case) in this file is the man.
 
**Couple's Data - Couple's Recode (CR)** 
This dataset has one record for every couple. It contains data for married or living together men and woman who both declared to be married (living together) to each other and with completed individual interviews (questionnaires). Essentially the file is the result of linking the two files previously described based on whom they both declared as partners. The unit of analysis (case) in this file is the couple.
 
**Children's Data - Children's Recode (KR)** 
This dataset has one record for every child of eligible women, born in the last five years. It contains the information related to the child's pregnancy and postnatal care and immunization and health. The data for the mother of each of these children is included. This file is used to look at child health indicators such as immunization coverage, vitamin A supplementation, and recent occurrences of diarrhea, fever, and cough for young children and treatment of childhood diseases. The unit of analysis (case) in this file is the children of women born in the last 5 years (0-59 months).
 
**Births' data - All Children's Recode (BR)** 
This dataset has one record for every child ever born of eligible women. Essentially, it is the full birth history of all women interviewed including its information on pregnancy and postnatal care as well as immunization and health for children born in the last 5 years. Data for the mother of each of these children is also included. This file can be used to calculate health indicators as well as fertility and mortality rates. The unit of analysis (case) in this file is the children ever born of eligible women.

**HIV Test data (AR)** 
This dataset has one record for every individual for which blood was drawn for HIV testing. In 2004 DHS began collecting blood for HIV testing but because of the sensitivity of the data instead of merging the results of HIV testing to the individuals a file that is distributed separately was created. This file can be linked to the household members (PR), the women (IR) or men files (MR).

Additionally, there are a number of files that can be associated to the files previously described but because of several reasons they are distributed separately.
 
**Wealth Index data (WI)** 
This dataset has one record for every household. Wealth Index analysis was introduced to DHS by the end of the 90’s. When the decision to include the wealth index as part of DHS was made, standard variables were introduced to the recode definition for both the household and individual questionnaires (HV270 and HV271 for households; V190 and V191 for women; and MV190 and MV191 for men). For previous surveys a file containing the score and the quintile variables, was created. Essentially wealth index files were created for all DHS surveys except surveys carried out as part of the first DHS phase. This file can be linked to any of the files described in the previous section.
 
**Height and Weight data according to WHO (HW)** 
This dataset has one record for every child measured for height and weight. In 2007 new child growth standards were introduced by WHO; in the past DHS used the NCHS/CDC/WHO reference. After the decision to adopt the new WHO standards was made, standard recode variables HC70 to HC73 and HW70 to HW73 were introduced to the recode definition to store the standard deviations of the new WHO child growth definition. Essentially all files using the DHS-5 recode structure have these variables. For previous surveys a file containing the same z-scores, was created. In early DHS phases only children of eligible women were measured. Starting with DHS-3 onwards all children under five listed in households interviewed have been measured. This file can be linked to the household members (PR), the children (KR) or the births (BR) files described above if height and weight was taken for children in the households. The file can only be linked to the children (KR) or birth (BR) files when only children of eligible women were measured for early DHS phases.

**Variable naming conventions.** Variables begin with one or two letters followed by one, two, or three digits and in some cases followed by a letter. Following is a list describing the general variable name conventions.

HVxxx  Household standard variables

Haxx  Anthropometry and anemia for women

HCxx Anthropometry and anemia for children

SHxxx Household, country-specific.

Vxxx  Women standard variables

Bxx Birth history

Mxx Pregnancy, postnatal care, and breastfeeding

Hxx Immunization and health

HWxx Anthropometry for children of eligible women

MMxx Maternal mortality (optional)

DVxx Domestic violence (optional)

Sxxx Women, country-specific

MVxxx Men standard variables

SMxx Men, country-specific

In this list “xx” represents digits, and the last one could be a letter. There are a handful of variables that do not fully adhere to this scheme (BORD, BIDX, MIDX, HWIDX, etc.), but at least the first letter will indicate where it belongs. The “xx” in the household, women, and men **country-specific** variables correspond to the actual question number in the country questionnaire. The maternal mortality (MMxx) and domestic violence (DVxx) variables will only be present if the modules were applied in the country.

If the calendar was used in the country, variables VCOL and VCAL will be present. These variables are part of a repeating record. VCOL is the calendar column number and VCAL is a string of 80 characters containing the actual calendar information.

The multiple or repeating records are placed one after the other on the record, with the maximum number of occurrences of each section being represented in every case. Each variable in a repeating section is placed immediately after the preceding variable of the same occurrence, such that all variables for occurrence 1 precede all variables for occurrence 2 of a section.

Multiple occurring variables and sections of data represent the main disadvantage to flat files. Each occurrence of every such variable must have its own name because statistical packages do not generally support the use of arrays or subscripts. For example, the third occurrence of the variable named MM11 would be named MM11$03 in SPSS, or MM11_03 in SAS and STATA.

**Some Useful DHS Links**

[Model questionnaires](http://dhsprogram.com/publications/publication-search.cfm?type=35)

[Model DHS Datasets](http://dhsprogram.com/data/Download-Model-Datasets.cfm)

[STATcompiler](http://www.statcompiler.com/)

[Final Country Reports](http://dhsprogram.com/publications/publications-by-type.cfm)

[DHS File Naming Convention](http://dhsprogram.com/data/File-Types-and-Names.cfm)

[DHS Recode Manual](http://dhsprogram.com/publications/publication-dhsg4-dhs-questionnaires-and-manuals.cfm).

[Guide to DHS Statistics](http://dhsprogram.com/publications/publication-dhsg1-dhs-questionnaires-and-manuals.cfm)

[DHS User Forum](http://userforum.measuredhs.com/)

[DHS Video Tutorials](https://www.youtube.com/playlist?list=PLagqLv-gqpTMx2Q10C_prJRnCtM55C0o8)

[Using DHS Data for Analysis](http://dhsprogram.com/data/Using-Datasets-for-Analysis.cfm)

[Merging DHS Datasets](http://dhsprogram.com/data/Merging-Datasets.cfm)
